# Editorial: Developing high-yielding plant cell bio-factories for high-value low-volume phytochemicals

**DOI:** 10.3389/fpls.2023.1281385

**Published:** 2023-09-25

**Authors:** Smita Srivastava, Milen I. Georgiev, Ramamoorthy Siva, Shyam Kumar Masakapalli

**Affiliations:** ^1^ Department of Biotechnology, Bhupat and Jyoti Mehta School of Biosciences, Indian Institute of Technology Madras, Chennai, India; ^2^ Laboratory of Metabolomics, Institute of Microbiology, Bulgarian Academy of Sciences, Plovdiv, Bulgaria; ^3^ Center of Plant Systems Biology and Biotechnology, Plovdiv, Bulgaria; ^4^ School of Bio Sciences and Technology, Vellore Institute of Technology, Vellore, India; ^5^ School of Biosciences and Bioengineering, Indian Institute of Technology Mandi, Kamand, Himachal Pradesh, India

**Keywords:** plant cells, biofactories, metabolic engineering, phytochemicals, process optimization

The development of sustainable plant cell bio-factories for mass production of value-added molecules holds the possibility to expand access to healthcare and meet the UN’s Sustainable Development Goals (SDGs) and the World Health Organisation’s (WHO) directive on the promotion of herbal medicines among the low-income population worldwide. The pharmaceutical potential of phytochemicals renders them potent drug candidates for the alleviation of several health disorders and the large-scale production of these metabolites is a pressing priority. Numerous phytochemicals have industrial significance in addition to their uses in medicine since they are also used in supplements, cosmetics, and other products. Most importantly, sustainable biomanufacturing based on plant cell bio-factories can promote the bioeconomy, which has become crucial for phasing out fossil resources and meeting the climate change mitigation goals via biodiversity conservation.

The main aim of this Research Topic was to bring together the current state of the art in the development of high-yielding plant cell bio-factories for economic feasibility in bioprocesses for the production of phytochemicals. Broadly, the Topic includes aspects of plant metabolic engineering, which involves deciphering the complex metabolic networks present in plants *via* flux balance analysis and further enhancing the production of phytochemicals using different metabolic engineering strategies such as overexpression, downregulation, transcription factor regulation, etc. Further, kinetic model-assisted process optimization at the bioreactor level can be used to achieve maximum productivity from plant cell bio-factories at a large scale.

To this effect, our Research Topic brings you an exciting consortium of seven research articles from across the globe. These articles underline the possibilities of utilizing various biotechnological approaches including metabolic engineering, metabolomics, transcriptomics, and machine learning algorithms for the sustainable production of high-value low-volume phytochemicals from plant biofactories. Hence, this Research Topic is an exemplification of constructive techniques for the production of high-value phytochemicals with immense beneficial properties. Crocins, originally from saffron (*Crocus sativus*) are extremely high-value soluble pigments used as supplements and colorants. Ahrazem et al. describe the metabolic engineering of crocin biosynthesis in *Nicotiana* species using single gene and multiple gene overexpression of carotenoid pathway genes for the enhanced production of crocins by nearly 3.5 fold, establishing a basis for the development of strategies that can be used for commercial exploitation of high-value products at a large scale. CRISPR/Cas9 has also been increasingly used to edit genomes for metabolic engineering purposes, among others. Göritzer et al. highlight the N-glycosylation pathway engineering in *Nicotiana tabacum* using CRISPR technology and pave the way for using it as a species of choice for scalable recombinant protein pharmaceuticals. With the increase in technological advancements, incorporating such tools/strategies for the production of phytochemicals is the need of the hour. In addition, using machine learning and multivariate analysis, plant hormones such as abscisic acid (ABA) and cytokinin isopentenyl adenosine (IPA) have been found to play a crucial role in determining centelloside content in *Centella asiatica* hairy roots as reported by Alcalde et al. Integrated transcriptomic and metabolomic approaches have been increasingly used as tools to understand several molecular mechanisms in plant metabolism. One such example is the use of salicylic acid (SA) elicitor to interpret transcriptomic and metabolic changes in callus cultures of *Pulsatilla chinesis*, rich in terpenoids with therapeutic potential as reported by Dong et al. A similar approach is used by Wang et al. to provide insights into key molecular and metabolic mechanisms involving transcription factor regulation for embryogenic potential maintenance in *Larix kaempferi*(Lamb.) Carr. with a focus on phenolic acids and flavonoids.

Plant cell bio-factories have also acted as platforms for the production of clinically important drugs. For instance, Exendin-4, a Glucagon-like peptide-1(GLP-1) for the treatment of type 2 diabetes, has been produced from *Nicotiana benthamiana*, where plant-produced exendin-4 was reported to be as effective as its chemically synthesized variant as reported by Akter et al. Murali et al. discuss the enhancement in the production of camptothecin, an important anti-cancer drug using rational metabolic engineering approaches in plant cells of *Nothapodytes nimmoniana*.

## Author contributions

SS: Conceptualization, Writing – original draft. MG: Writing – review & editing. RS: Writing – review & editing. SM: Writing – review & editing.

